# Biofunctionalization of Poly(lactide-*co*-glycolic acid) Using Potent NorA Efflux Pump Inhibitors Immobilized on Nanometric Alpha-Zirconium Phosphate to Reduce Biofilm Formation

**DOI:** 10.3390/ma14030670

**Published:** 2021-02-01

**Authors:** Monica Pica, Nicla Messere, Tommaso Felicetti, Stefano Sabatini, Donatella Pietrella, Morena Nocchetti

**Affiliations:** 1 Dipartimento di Scienze Farmaceutiche, Università di Perugia, Via del Liceo, 1, 06123 Perugia, Italy; monica.pica@unipg.it (M.P.); niclamessere1@gmail.com (N.M.); tommaso.felicetti90@gmail.com (T.F.); 2 Dipartimento di Medicina, Università di Perugia, Piazzale Gambuli, 1, 06132 Perugia, Italy; donatella.pietrella@unipg.it

**Keywords:** zirconium phosphate, PLGA, Efflux pump inhibitors, biofilm inhibition, composites

## Abstract

Polymeric composites, where bioactive species are immobilized on inorganic nanostructured matrix, have received considerable attention as surfaces able to reduce bacterial adherence, colonization, and biofilm formation in implanted medical devices. In this work, potent in-house *S. aureus* NorA efflux pump inhibitors (EPIs), belonging to the 2-phenylquinoline class, were immobilized on nanometric alpha-zirconium phosphate (ZrP) taking into advantage of acid-base or intercalation reactions. The ZrP/EPI were used as filler of poly(lactide-*co*-glycolic acid) (PLGA) to obtain film composites with a homogeneous distribution of the ZrP/EPI fillers. As reference, PLGA films loaded with ZrP intercalated with thioridazine (TZ), that is recognized as both a NorA and biofilm inhibitor, and with the antibiotic ciprofloxacin (CPX) were prepared. Composite films were characterized by X-ray diffraction, scanning electron microscopy, and thermogravimetric analysis. The ability of the composite films, containing ZrP/EPI, to inhibit biofilm formation was tested on *Staphylococcus aureus* ATCC 29213 and *Staphylococcus epidermidis* ATCC 12228, and it was compared with that of the composite loaded with ZrP/TZ. Finally, the antibacterial activity of CPX intercalated in ZrP was evaluated when used in combination with ZrP/EPI in the PLGA films.

## 1. Introduction

The human health and the longevity have gained huge improvements from the antibiotic discovery. However, nowadays, several pathogenic bacteria have developed antibiotic resistance, therefore, the humanity will face, in the 21st century, an important challenge that is to develop new therapies to treat bacterial infections [1]. The antibiotic resistance has been caused by incorrect behaviours, such as the inappropriate antibiotic use, extensive use in agricultural and veterinary sectors and ageing populations and the pathogens diffusion has been favoured by the growing global travel and migration [2].

Several bacteria belonging to both Gram-positive and Gram-negative families showhigh levels of resistance to different antibiotic classes, among them are *Staphylococcus aureus*, *Staphylococcus epidermidis*, *Enterococcus faecium*, *Klebsiella pneumoniae*, *Escherichia coli*, *Pseudomonas aeruginosa*, *Acinetobacter baumannii* [3].

Mechanisms of drug resistance include drug inactivation or alteration by bacterial enzymes, alteration of target sites that avoids their recognition by antimicrobial agents, reduced intracellular drug accumulation by diminishing the number of protein channels on the bacterial outer membrane and/or by the presence of efflux pumps (EPs), and biofilm formation [4]. As concern the therapies, for a long time, the medicinal chemistry has focused its attention on the design and synthesis of single target antibiotics, however, the development of the resistance to these drugs seems very fast and not sustainable. Therefore, the research has moved towards multitarget approaches using multitarget compounds, antibiotic combinations, and coadministration of antibiotics with permeabilizers, antibiotic sensitizers, β-lactamase inhibitors, biofilm inhibitors, and efflux pump inhibitors (EPIs) [5]. Among these, the basal expression and much more the overexpression of EPs in bacteria is widely studied because it is connected with an increasing rate of new mutations, the horizontal transfer of antibiotic resistance [6,7,8], and the biofilm formation [9].

Biofilm formation over implanted medical devices is the most common cause of infections and represent the most severe complication associated to the use of biomaterials and a relevant social and economic cost for the patient and for the national health system [10,11]. Bacteria in planktonic form colonize the surface and in the first stage of biofilm formation, they are still susceptible to the antimicrobial action. In the next few hours, the microbial cells increase in mass and bulk to form microcolonies embedded in a self-produced polymer matrix; finally, the biofilm grows to form a mature biofilm that is resistant to systemic antibiotic therapies [12,13]. Great attention is paid, to overcome this problem, on the functionalization of biomaterial surfaces with non-antibiotic antibacterial substances in order to reduce bacterial adherence, colonization, and biofilm formation [14] and, at the same time, avoid the development of multidrug resistant bacterial strains. In this connection, the research is focused on the growing or deposition of composites obtained by the combination of inorganic materials with bioactive species on biomaterial surface [15,16,17]. Bioactive composites have been prepared by immobilizing antibacterial agents, such as Ag, Au, Cu, TiO_2_, and ZnO [18,19] on hydroxyapatite, titania nanotubes, calcium silicate [20], bioactive glasses, and silica-based MCM-41 [21]. Moreover, implants with anti-infective properties have been obtained upon the surface modification with nitric oxide (NO)-releasing materials [22].

**Figure 1:**
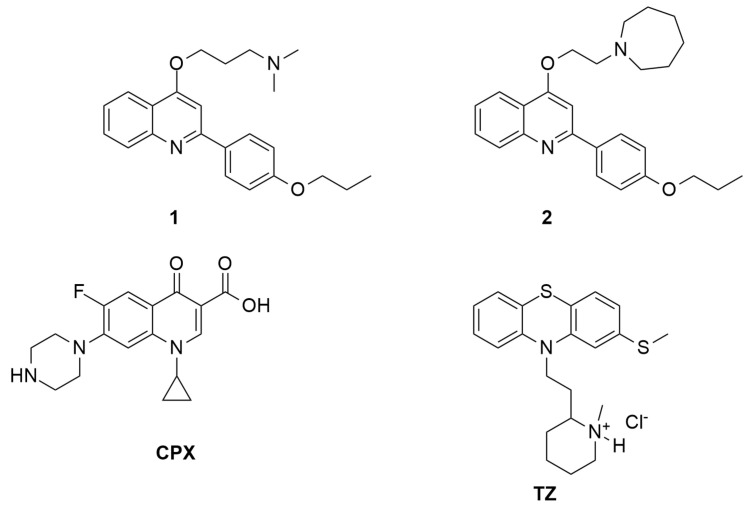
Chemical structure of *S. aureus* NorA efflux pump inhibitors (EPIs) **1** and **2**, ciprofloxacin (CPX), and thioridazine (TZ).

The use of polymeric nanocomposites as coating of biomaterials is a valuable strategy to immobilize the active species on the biomaterial surface [23]. Particular attention has been focused on poly(lactide-*co*-glycolic acid) (PLGA) since it is biocompatible, biodegradable, and is able to deliver antibiotics [24] and growth factors to enhance bone healing in orthopaedic applications [25]. Moreover, nanocomposites based on PLGA and metal-based nanostructures have shown antimicrobial activity and improved mechanical properties [26,27]. The preparation of PLGA composites containing EPIs as synthetic molecules and even inorganic particles as ZnO [28] or some essential oils [29] should be an innovative strategy to prepare films that reduce or reverse bacterial resistance to antibiotics. This work aimed to the preparation of film nanocomposites based on PLGA loaded with nanometric alpha-zirconium phosphate (ZrP) functionalized with potent in-house *S. aureus* NorA EPIs, belonging to the 2-phenylquinolines class (**1** and **2** of Figure 1) [30]. ZrP is a layered material widely used as drug delivery system due to its excellent biocompatibility and biological inertness [31,32]. The presence of acidic OH groups on the ZrP surface allows the intercalation of basic species such as **1** and **2** *S. aureus* NorA EPIs and even releases them upon a pH changes or gradient concentration. Moreover, the nanometric dimensions, ranging from 30 to 200 nm, allow to obtain polymeric composites with a homogeneous distribution of the functionalized filler. Composite films constituted by PLGA and ZrP functionalized with **1** and **2** were prepared by solvent casting and characterized by X-ray diffraction, scanning electron microscopy, and thermogravimetric analysis. The ability of the composite films to inhibit biofilm formation was tested on *S. aureus* ATCC 29213 and *S. epidermidis* ATCC 12228. The activity of film composites containing **1** and **2** was compared with that of a composite loaded with ZrP intercalated with thioridazine (TZ) (Figure 1), that is recognized as both a *S. aureus* NorA and a biofilm inhibitor but only at toxic concentrations [33]. Moreover, the antibacterial activity of ciprofloxacin (CPX) (Figure 1) was evaluated when used in combination with *S. aureus* NorA EPIs in the composite films.

## 2. Experimental

### 2.1. Chemicals

Zirconyl propionate (ZrO_1.27_(C_2_H_5_COO)_1.46_, MW = 218 g/mol) was supplied by MEL Chemicals, England. Ethyl acetate (EA) was purchased from Carlo Erba and poly(lactic-*co*-glycolic acid) (85:15 lactic acid:glycolic acid, average MW = 150,000 g/mol, hereafter, PLGA) from PolySciTech (West Lafayette, IN, USA). Ciprofloxacin (MW = 331.4 g/mol, hereafter labelled as CPX), thioridazine hydrochloride (MW = 407.0 g/mol, hereafter labelled as TZ), and all other reagents were purchased from Aldrich, and used without any further purification. Muller Hinton Broth (MHB), Mueller Hinton Agar (MHA), sucrose, and Crystal-violet were purchased from Sigma-Aldrich (Merck).

### 2.2. Synthesis of NorA Efflux Pump Inhibitors: **1** and **2**

The *S. aureus* NorA efflux pump inhibitors (EPIs) **1** and **2** were synthetized as reported in reference [30].

### 2.3. Synthesis of Nanocrystalline ZrP

A gel of nanocrystalline ZrP (hereafter, ZrP) in propanol was synthesized according to [34]. A 0.35 M solution of zirconyl propionate in propanol was prepared by dissolving 763 mg of zirconyl propionate in 10 mL of propanol. At this solution 1.2 mL of 14.8 M solution of phosphoric acid was added (H_3_PO_4_/Zr molar ratio = 5) at room temperature and under stirring. The gel, formed after a few minutes, was washed three times with propanol in order to remove the excess of phosphoric acid and by-products. The weight percentage of ZrP in the gel, determined by drying in an oven at 80 °C, was about 8%.

### 2.4. Preparation of the Composite of ZrP and Ciprofloxacin (ZrP/CPX)

A 0.05 M solution of ciprofloxacin hydrochloride was prepared by dissolving 220 mg of CPX in 13.3 mL of 0.05 M HCl. The solution was added to 2.5 g of ZrP gel, so that the CPX/ZrP molar ratio was about 1. The mixture was left under stirring at room temperature for 24 h, then centrifuged, washed two times with water, and finally, dried at room temperature under vacuum, over P_2_O_5_.

### 2.5. Preparation of the Composite of ZrP and **1** (ZrP/**1**)

A solution of **1** was prepared by dissolving 100 mg of solid in 2 mL of propanol. The solution was added to 2.5 g of ZrP gel, so that the **1**/ZrP molar ratio was about 0.3. The mixture was left under stirring at 80 °C for 2 days, then centrifuged, washed two times with propanol, and finally, dried in an oven at 60 °C.

### 2.6. Preparation of the Composite of ZrP and **2** (ZrP/**2**)

A solution of **2** was prepared by dissolving 42 mg of solid in 2 mL of propanol. The solution was added to 2.5 g of ZrP gel, so that the **2**/ZrP molar ratio was about 0.14. The mixture was left under stirring at 80 °C for 2 days, then centrifuged, washed two times with propanol, and finally, dried in an oven at 60 °C.

### 2.7. Preparation of the Composite of ZrP and Thioridazine (ZrP/TZ)

The preparation of ZrP/TZ composite material was reported in the Supporting Information (Figures S1 and S2).

### 2.8. Preparation of the PLGA Composite Films

About 200 mg of PLGA was solubilized in 6.7 mL of ethyl acetate at room temperature. The solution was cast into a Petri dish (diameter = 6 cm), and the solvent was evaporated at room temperature (Film A).

Composite films of PLGA containing 10 and 20 wt% of ZrP were also prepared. First, ZrP gel in propanol was washed two times with ethyl acetate, thus obtaining a gel of ZrP in ethyl acetate. Suitable amounts of the ZrP gel in ethyl acetate were added to the polymer solution and the mixture were left under stirring at room temperature for 24 h, then sonicated for 3 min, cast into a Petri dish (diameter = 6 cm), and finally, dried at room temperature. The composites with 10 and 20 wt% of ZrP will be, hereafter, labelled as Film B and Film C, respectively.

Using a similar procedure, composite films of PLGA and ZrP/CPX, ZrP/**1**, and ZrP/**2** (Film D, Film E, and Film F, respectively), with filler loadings in the range of 10–20 wt%, were prepared.

Composite films of PLGA containing two different composites, specifically ZrP/CPX + ZrP/**1** (Films G, H) and ZrP/CPX + ZrP/**2** (Films I, J) were also prepared. The amount of each ZrP/X composite was chosen in order to have similar amount of CPX in all films (around 2–3 wt%), while the amounts of the *S. aureus* NorA EPIs **1** or **2** were chosen in order to have films with CPX/EPI molar ratios ≈ 1 (films H and J) and 3 (films G and I).

The preparation of the PLGA film containing ZrP/TZ (Film K) is reported in the Supporting Information.

All the PLGA composite films prepared in the present work are listed in Table 1.

### 2.9. In Vitro Static Biofilm Assay

The biofilm inhibition activity of the films reported in Table 1 was tested on the microbial strains *Staphylococcus aureus* ATCC 29213 and *Staphylococcus epidermidis* ATCC 12228 maintained in Mueller Hinton agar (MHA).

The in vitro static biofilm assay was carried out by changing properly the method described in references [35,36]. The films were cut in disk shape of 6 mm diameter, weighted, sterilized under UV ray for 30 min and placed into the wells of 96-well microplates. A single colony of test strains was grown for 24 h at 37 °C in Mueller Hinton Broth (MHB). The inocula were obtained by diluting the cultures of *S. aureus* or *S. epidermidis* with Mueller Hinton broth (MHB) supplemented with 2% sucrose (MHB-S) (dilution = 1:100, corresponding to 10^6^–10^7^ CFU mL^−1^), and then, 100 μL of this was inoculated in each well with 100 μL of MHB-S. After incubation at 37 °C for 24 h in static conditions, the biofilm grown in each well was washed twice with 200 μL of distilled water and treated with 100 μL of 0.4% crystal violet for 30–45 min. To remove the excess of crystal violet, the wells were washed four times with distilled water. Then, the wells were treated with 200 μL of 95% ethanol for 45 min. The absorbance of the destaining ethanol solution was measured at 570 nm (OD_570_) in a microplate reader (TECAN) after having transferred a portion of solution (100 μL) to a well of a new plate.

Film K was used as positive control and Films A, B, and C as negative control. Biofilm assays were performed in triplicate, and the data were expressed as mean ± SD. Differences between groups were compared using the Student’s *t*-test (two-tailed). A *p*-value of <0.05 was considered significant.

### 2.10. Instrumentation

The X-ray diffraction (XRD) patterns were performed by the Philips X’PERT PRO MPD diffractometer (PANalytical, Royston, United Kingdom) operating at 40 KV and 40 mA, using a X’Celerator detector (PANalytical, Royston, United Kingdom). The used radiation is Cu Kα and step scanning method (step size 0.03 2 θ°, step scan 30 s) was used to record the spectra.

The thermogravimetric analysis (TG) was carried out by the system TG-DTA Netzsch STA 490C (Netzsch, Selb, Germany). The experimental conditions were heating rate of 10 °C/min and air flow of 30 mL/min.

The morphology of the samples was investigated with a scanning electron microscope (FE-SEM, FEG LEO 1525) (Zeiss, Jena, Germany). FE-SEM micrographs were collected on the fractured surfaces of PLGA film. The films were fractured in liquid nitrogen and then the samples were anchored on a stub and sputter coating with chromium for 20 s.

## 3. Results and Discussion

With the aim to select the most suitable inorganic support for the immobilization of **1**, **2**, CPX, and TZ (generally indicated hereafter as X), we considered the probable interactions that could occur between the selected active molecules and the inorganic support. The presence of amino groups in X, able to interact with protogenic groups, suggested that zirconium phosphate is the best choice as inorganic support, due to the presence of the POH groups that could interact with the amino groups of the guest molecules through acid-base (as **1** and **2**) or ion exchange (as CPX and TZ hydrochlorides) reactions. We selected a nanocrystalline ZrP material, since it can be directly prepared in the form of solvent intercalated packed layers that facilitates the insertion in the interlayer region of large molecules as the bioactive species used in the present paper.

### 3.1. Characterization of the ZrP/X Composite Powders

ZrP/X powder samples were analysed by thermogravimetry (TG) and X-ray diffraction (XRD). The characterization of the ZrP/TZ material was reported in the Supporting Information. In the following paragraphs, the characterization of ZrP/X, X = CPX, **1**, **2**, is reported.

**Figure 2:**
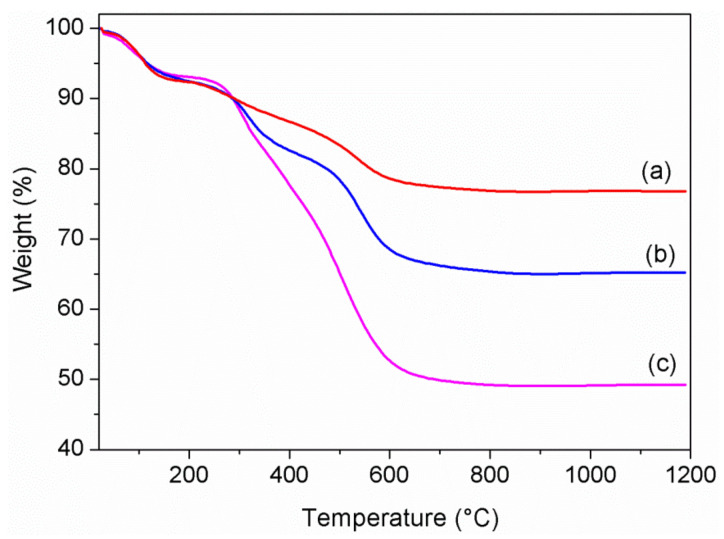
Thermogravimetric curves of alpha-zirconium phosphate (ZrP)/**1** (**a**), ZrP/**2** (**b**), and, ZrP/CPX (**c**).

First, TG analysis was carried out under air flow in the temperature range of 20–1200 °C, in order to verify and calculate the uptake of bioactive species. The TG profiles of the ZrP/X composites are shown in Figure 2.

It is known that monohydrated α-ZrP exhibits a first weight loss (≈6%) below 150 °C due to crystallization water, and a second loss (≈6%) above 200 °C due to condensation water, with formation of cubic zirconium pyrophosphate at about 700 °C [31]. All the composites showed a weight loss in the range of 200–1200 °C greater than 6%, indicating the occurred uptake of bioactive molecules by ZrP, which are decomposed in that range of temperature. By assuming the following composition for the composites, Zr(HPO_4_)_2_*n*X*m*S, where X is the bioactive species and S is the solvent, the values of *n* and *m* were calculated by the TG curves, and the resulting values were reported in Table 2.

**Figure 3:**
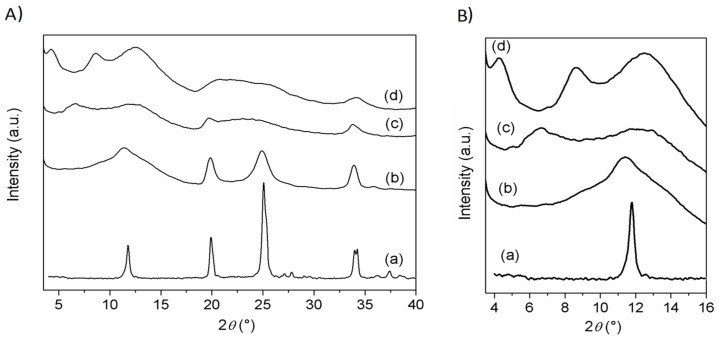
XRD powder patterns of (**A**) ZrP (a), ZrP/**1** (b), ZrP/**2** (c), and ZrP/CPX (d). (**B**) Enlargement in the 4–16° 2θ region of (**A**).

XRD patterns of ZrP/CPX, ZrP/**1**, and ZrP/**2** powders, together with that of pristine ZrP, are shown in Figure 3.

ZrP pattern shows the typical peaks of the α-type structure, with the basal peak at 11.6° 2θ (*d* = 7.6 Å) and that associated with the Zr−Zr separation (5.3 Å) within the α-type layer at 33.8° 2θ. The patterns of the composites are characterized by broader and less intense peaks, with respect to pristine ZrP. The pattern of ZrP/**1** resembles that of pristine ZrP, suggesting that **1** is not intercalated, but simply adsorbed on the external surface of the ZrP particles. Differently, the patterns of ZrP/**2** and ZrP/CPX exhibited broad peaks at angles lower than 11.6° 2θ (see Figure 3B), indicating an enlargement of the interlayer distance due to the intercalation of guest molecules. Specifically, for ZrP/**2**, a peak at 6.65° 2θ (*d* = 13.3 Å) was observed, while ZrP/CPX shows a first-order peak at 4.4° 2θ (*d* = 20 Å) and a second-order peak at 8.7° 2θ (*d* = 10 Å). In both cases, a broad peak in the 2θ range of 10–14° was present, suggesting the copresence of an unintercalated ZrP phase.

### 3.2. Characterization of the Composite Films A–F

**Figure 4:**
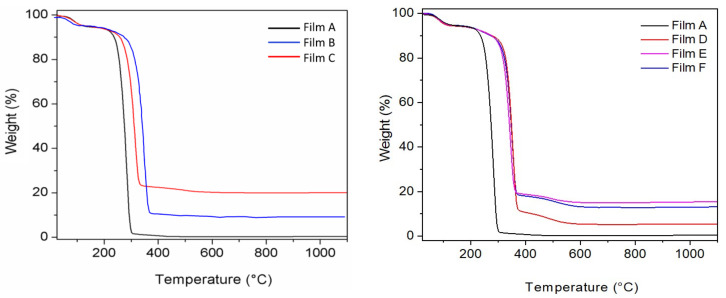
TG profiles of poly(lactide-*co*-glycolic acid) (PLGA)-based films: pure PLGA (film A), PLGA_ZrP (films B and C), PLGA_ZrP/CPX (film D), PLGA_ZrP/**1** (film E), and PLGA_ZrP/**2** (film F).

The above ZrP/X composites were used to prepare composite films based on PLGA. The gels of both ZrP and ZrP/X were first washed with the same solvent used to solubilize PLGA (ethyl acetate). In order to determine the effective amount of filler dispersed in the polymer matrix, the composites films were analysed by thermogravimetry. The characterization of the PLGA composite film containing ZrP/TZ was reported in the Supporting Information. Figure 4 shows the TG profiles of all other PLGA composite films.

All curves exhibited a first weight loss below 200 °C, due to solvent elimination, and a second weight loss in the temperature range of 200–1100 °C, due to the decomposition of the organic components (polymer and bioactive molecules), with formation of ZrP_2_O_7_ for the PLGA films filled with ZrP or ZrP/X. It is noteworthy that all composites start to decompose at temperatures higher than that of the pure polymer. In particular, by considering the temperature at which a 20% of weight loss occurred, a temperature shift, ΔT, of about 30 °C was observed in the presence of pure ZrP, while ΔT was in the range of 63–74 °C in the presence of ZrP/X, suggesting that the presence of the bioactive molecules further improves the thermal behaviour of the corresponding composites, probably acting as compatibilizers between the polymer and the inorganic filler. From the residual weights at 1100 °C of the TG curves C–F of Figure 4 and from data of Table 2, the effective composition of the composite films was calculated and the results are shown in Table 3.

**Figure 5:**
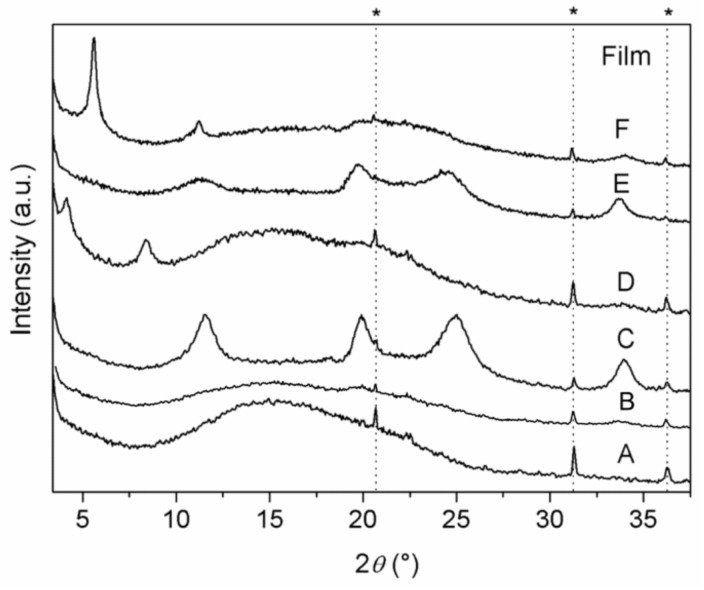
XRD patterns of Films A–F. Peaks labelled with the asterisk (*) belong to the aluminium film holder.

XRD patterns of the polymeric Films A–F are shown in Figure 5. Pure PLGA Film (A) exhibited the typical pattern of amorphous polymers, with a halo in the 2 θrange of 10–25°. The lack of reflections attributable to the filler in Film B suggests the formation of a nanocomposite in which most of the filler is exfoliated. On the other hand, broad diffraction peaks, due to the presence of the fillers, are observed in the patterns of the composite Films (C–F), suggesting the formation of phase-separated microcomposites and/or intercalated composites, even though a partial filler exfoliation cannot be excluded. Specifically, pattern of Film C (containing ZrP), as well as that of Film E (containing ZrP/**1**), shows broad typical peaks typical of low crystalline ZrP, indicating the formation of phase-separated microcomposites. About patterns D (containing ZrP/CPX) and F (containing ZrP/**2**), diffraction peaks due to the fillers are observed at 4.1° and 5.6° 2 θ, corresponding to interlayer distances of 21.2 and 15.7 Å, respectively; these values are slightly larger than those of ZrP/CPX and ZrP/**2** intercalation compounds, indicating an enlargement of the interlayer distance likely due to the diffusion of polymer chains in the interlayer region to some extent.

**Figure 6:**
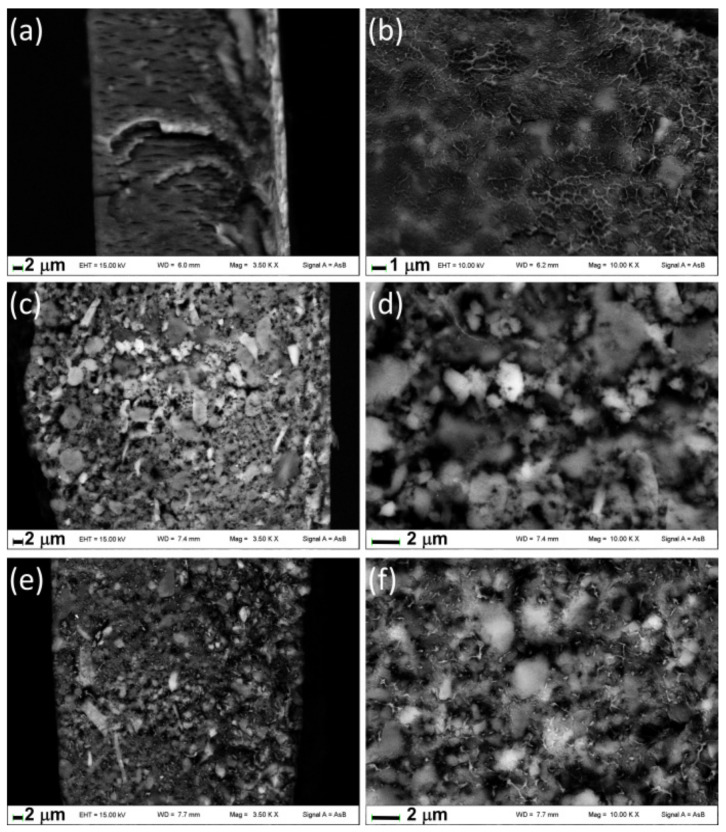
FE-SEM images of fractured surface of Films D (**a**,**b**), E (**c**,**d**), and F (**e**,**f**).

Figure 6 shows the images of fractured surfaces of films containing ZrP/X. Note that the fractured surface of films presents holes due to the effect of the electron beam of the microscope that causes the burning of the polymeric component; this fact did not allow us to collect images at higher magnification than those reported in Figure 6. The fracture of Film D (Figure 6a,b) appears uniform and the inorganic particles, revealed from the brightest zones, are finely distributed in the polymeric matrix due to a probable diffusion of the organic chains in the interlayer region as the XRD pattern suggests. Conversely, aggregates of inorganic particles are well evident in Films E and F showing the formation of microcomposites; even if in Film F, a certain degree of polymer intercalation is detected from XRD it is not so extended to give rise to complete disaggregation of the ZrP/**2**.

### 3.3. Characterization of the Composite Films G–J

In order to evaluate the possible synergistic effect between the *S. aureus* NorA EPIs (**1** and **2**) and the antibacterial CPX, films containing two different composite materials, specifically ZrP/CPX + ZrP/**1** (films G and H) and ZrP/CPX + ZrP/**2** (films I and J), were also prepared.

The amount of each ZrP/X composite was chosen in order to have similar amount of CPX in all films (around 2–3 wt%), while the amounts of the inhibitors **1** or **2** were chosen in order to have CPX/inhibitor molar ratios ≈1 (films H and J) and ≈3 (films G and I).

The amounts of each ZrP/X composite (expressed as g per 100 g PLGA) used for the preparation of the films, as well as the percentage of CPX, **1,** and **2** in the final composite films, are reported in Table 4.

**Figure 7:**
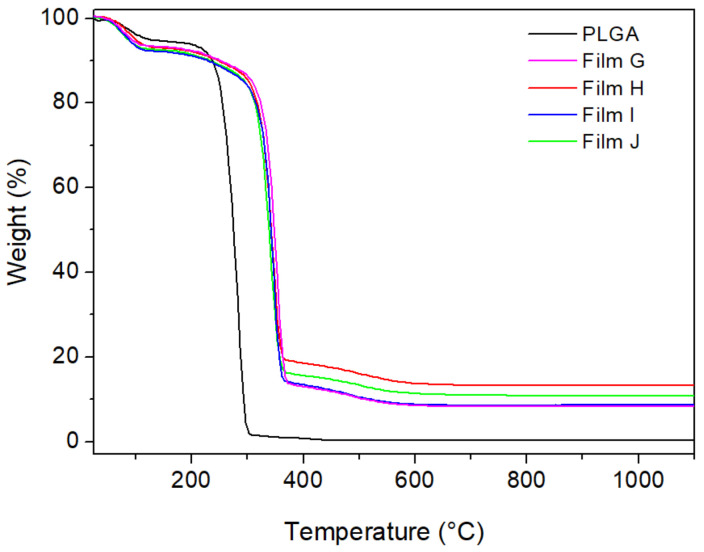
TG curves of PLGA films containing: ZrP/CPX + ZrP/**1** (films G and H) and ZrP/CPX + ZrP/**2** (films I and J).

Figure 7 shows the TG curves of Film A and Films G–J. As also found for Films D–F, the initial decomposition of the polymer matrix was shifted of about 65–70 °C toward higher temperatures in the presence of the fillers. From the residual mass at 1100 °C, the wt% of ZrP in the composites was calculated, resulting in quite good agreement with the values calculated according to the data reported in Table 4, with a maximum relative difference of 12%.

**Figure 8:**
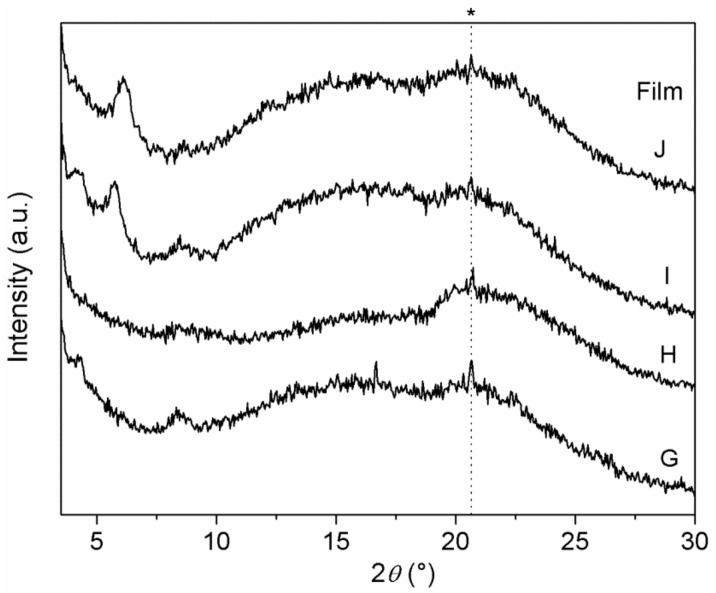
XRD patterns of PLGA composite films containing: ZrP/CPX + ZrP/**1** (films G and H) and ZrP/CPX + ZrP/**2** (films I and J). Peaks labelled with the asterisk belong to the aluminium film holder.

XRD patterns of Films G–J are shown in Figure 8. In comparison with Films D–F, containing a single ZrP/X composite, peaks in Figure 8 are broader and less intense.

Interestingly, Film H resulted to be basically amorphous, suggesting that both ZrP/CPX and ZrP/**1** underwent a significant exfoliation within the polymer matrix. On the contrary, peaks due both to ZrP/CPX (at 4.2° and 8.4° 2θ) and to ZrP/**2** (at 5.7° 2θ) are observed in pattern of Film I.

### 3.4. Evaluation of the Biological Activity of the Composite Films

In the present paper, we selected two Gram-positive bacteria, *S. aureus* ATCC 29213 and *S. epidermidis* ATCC 12228, known for their ability to form biofilms on prostheses and catheters, to test the biofilm inhibition capability of PLGA composite films loaded with the bioactive species ZrP/X. The biological activity of the composite films was determined as reported in the experimental part. Films A, B, and C were tested as negative controls, while the PLGA film containing 25 wt% ZrP/TZ (film K) was tested as positive control, since TZ is known to be an inhibitor of NorA efflux pump.

**Figure 9:**
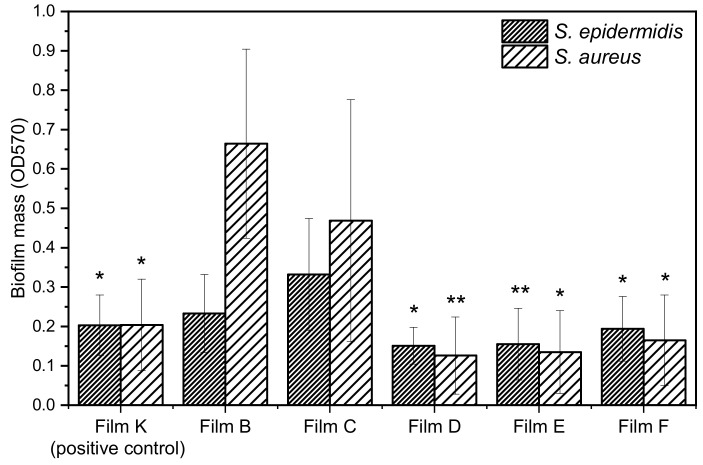
Effect of ZrP/**1** and ZrP/**2** loaded in PLGA film (Film E and Film F, respectively) on the biofilm formation of *S. aureus* ATCC 29213 and *S. epidermidis* ATCC 12228. Film containing ZrP/TZ (Film K) was used as positive control. Film containing ZrP/CPX (Film D) was used to evaluate its ability to inhibit biofilm. Data represent the mean ± SD of two independent experiments performed in triplicate. * *p* < 0.05; ** *p* < 0.01.

Figure 9 reports the biofilm mass grown on the composite films. Films B and C are not able to inhibit the growth of biofilm attesting that ZrP and PLGA do not exert any type of activity and they are referred as negative control. Conversely, films containing the species **1** and **2** are active against both biofilm formation of *S. aureus* and *S. epidermidis* and their activity is comparable to those of films loaded with ZrP/TZ and ZrP/CPX.

The antibiofilm activity can be also discussed consideringthe percentage of biofilm inhibition of the composite films. This was evaluated by comparing the biofilm mass grown on the composite films containing the bioactive species (films D, E, F, and K) with that grown on the PLGA films containing ZrP (films B and C), according to the following formula:

(1) $$\%\;\mathrm{of}\;\mathrm{biofilm}\;\mathrm{inhibition}\;=\;\left[1\;-\;\left({\mathrm{OD}}_{570}\;\mathrm{of}\;\mathrm{cells}\;\mathrm{treated}\;\mathrm{with}\;\mathrm{PLGA}-\mathrm{ZrP}/X/{\mathrm{OD}}_{570}\;\mathrm{of}\;\mathrm{cells}\;\mathrm{treated}\;\mathrm{with}\;\mathrm{PLGA}-\mathrm{ZrP}\right)\right]\;\times \;100$$

OD_570_ is the absorbance at 570 nm measuredafter staining with crystal violet. Films with similar filler loading were compared: specifically, Film D was compared with Film B, while Films E, F, and K were compared with film C. The percentages of biofilm inhibition for *S. epidermidis* and *S. aureus*, grown on the PLGA-based films, is shown in Table 5.

All composite films are more active toward *S. aureus* than toward *S. epidermidis*. Moreover, Film E, containing **1**, resulted the most active toward *S. epidermidis* (≈53% of biofilm inhibition)*,* while Film D, containing CPX, was the most active toward *S. aureus* (≈81% of biofilm inhibition), followed by Film E with ≈71% of biofilm inhibition. These films also resulted significantly more active than the positive control (film K).

**Figure 10:**
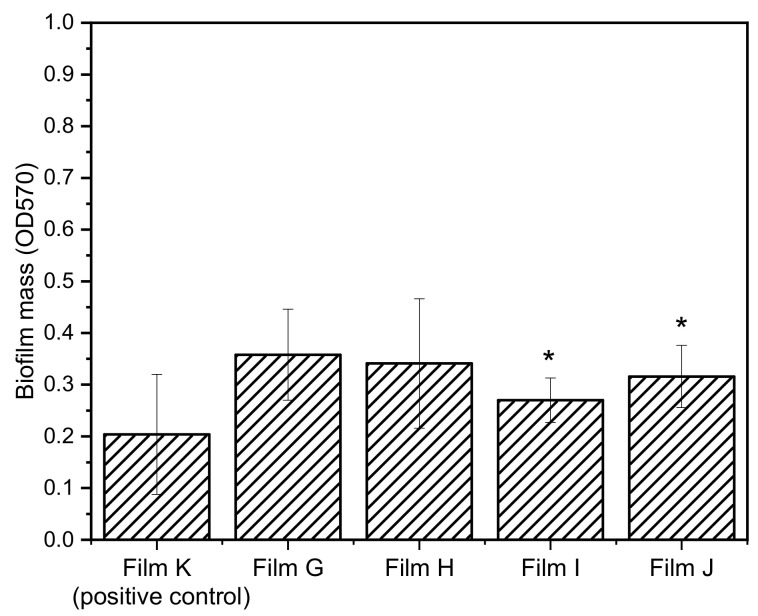
Biofilm mass of *S. aureus* ATCC 29213 grown on PLGA films loaded both with ZrP/**1** and ZrP/CPX (Film G and Film H) and both with ZrP/**2** and ZrP/CPX (Film I and Film J). The films differ for the CPX/EPI molar ratio (see Table 4). Film containing ZrP/TZ (Film K) was used as positive control. Data represent the mean ± SD of two independent experiments performed in triplicate. * *p* < 0.05.

In order to evaluate the possible synergistic effect between the inhibitors of the efflux pump NorA and the antibacterial CPX, films containing both CPX and **1** (films G and H) and CPX and **2** (Films I and J) were prepared. Since films D–F resulted to be more active toward *S. aureus*, the biofilm growth inhibition of films G–J was tested only toward *S. aureus*. The biofilm mass growth and percentages of biofilm inhibition for *S. aureus* are shown in Figure 10 and Table 6, respectively.

Generally speaking, films G–J exhibited lower capability of inhibiting the biofilm growth with respect to the films containing a single bioactive molecule, resulting even lower than that of the positive control (Films G, H, I, and J). Interestingly, the films containing (ZrP/CPX + ZrP/**2**) resulted to be more active than those containing the combination ZrP/CPX + ZrP/**1** showing that **2** in association with CPX is more active than **1** derivative. Moreover, by comparing composite films containing the same inhibitor (G–H and I–J), Films G and I, with an excess of CPX with respect to the inhibitors, exhibited the best performances.

## 4. Conclusions

The particular features of nanometric ZrP, such as the presence of POH groups and the reduced dimension of the particles (30–200 nm), combined with the possibility to prepare it in the form of solvent intercalated packed layers were exploited to efficiently immobilize **1** and **2** *S. aureus* NorA EPIs, TZ, and CPX. The compounds obtained were used as fillers of PLGA and the composites were processed as films. The XRD and SEM images, collected on the fractured surface, suggested that CPX in ZrP acts also as compatibilized between the inorganic component and the polymer by promoting the diffusion of polymer chains in the interlayer region of the ZrP and by forming an intercalated composite. Conversely, when **1** and **2** are immobilized in ZrP, the formation of microcomposites is obtained. The biofunctionalized PLGA films were tested for their biofilm inhibition capability.Films containing the species **1** and **2** are active against both biofilm formation of *S. aureus* and *S. epidermidis* and their activity is comparable to those of films loaded with ZrP/TZ and ZrP/CPX. Films containing both CPX and **1** and CPX and **2** were investigated in order to study the possible synergistic effect between the inhibitors of the efflux pump NorA and the antibacterial CPX. The biofilm growth inhibition was tested toward *S. aureus,* and it was found that **2** in association with CPX is more active than **1** derivative. Future investigation will be devoted to study the biofilm growth inhibition of films containing **1** and **2** by adding CPX to the bacterial cultures.

## Figures and Tables

**Table 1:** Poly(lactide-*co*-glycolic acid) (PLGA)-based polymeric films prepared in the present work.

PLGA Film	FILLER
A	–
B–C *	ZrP
D	ZrP/CPX
E	ZrP/1
F	ZrP/2
G–H *	ZrP/CPX + ZrP/1
I–J *	ZrP/CPX + ZrP/2
K	ZrP/TZ

* Different for the amount of the filler (wt%), see Table 3.

**Table 2:** Composition of the ZrP/X composite materials.

Bioactive Molecule		Zr(HPO_4_)_2_*n*X*m*S	
X	wt%	n	m *
1	10.6	0.10	0.5
2	22.9	0.23	0.6
CPX	40.6	0.66	1.9

* S = water for CPX; S = propanol for **1** and **2**.

**Table 3:** Composition of the PLGA_ZrP/X composite films.

Film	X	ZrP/X (wt%)	X (wt%)
B	–	6	–
C	–	20	–
D	CPX	11	5.1
E	1	20	2.3
F	2	19	4.2

**Table 4:** Composition, expressed as g/100 g PLGA, of the Films G–J.

Film	ZrP/CPX	ZrP/1	ZrP/2	CPX	1	2	CPX/1 Molar Ratio	CPX/2 Molar Ratio
G	8.3	11.1	–	2.8	1.0	–	3.1	–
H	6.5	22.8	–	2.0	1.9	–	1.2	–
I	8.3	–	6.0	2.9	–	1.2	–	2.9
J	7.5	–	17.5	2.4	–	3.2	–	0.93

**Table 5:** Percentage of biofilm inhibition of the indicated films.

	Film K	Film D	Film E	Film F
*S. epidermidis* ATCC 12228	38.9	35.2	53.3	41.6
*S. aureus* ATCC 29213	56.5	81.0	71.2	64.8

**Table 6:** Percentage of biofilm inhibition of the indicated films.

	Film K	Film G	Film H	Film I	Film J
*S. aureus* ATCC 29213	57	40	19	55	25

## Data Availability

The data presented in this study are available in this article and in the supplementary material.

## Supplementary Materials

The following are available online at https://www.mdpi.com/1996-1944/14/3/670/s1, Figure S1: TG profile (a) and XRD (b) of ZrP/TZ, Figure S2: TG profile (a) and XRD (b) of PGLA (Film A) and PLGA_ZrPTZ (Film K).

## Abbreviations

PLGA	poly(lactide-*co*-glycolic acid)
EPs	efflux pumps
EPIs	efflux pump inhibitors
ZrP	alpha-zirconium phosphate
TZ	thioridazine
CPX	ciprofloxacin
X	generally indicates CPX, TZ, **1**, and **2**
MHB	Muller Hinton Broth
MHA	Mueller Hinton Agar
MHB-S	Mueller Hinton broth supplemented with 2% sucrose
