# Supplementary material for: Biofunctionalization of Poly(lactide-co-glycolic acid) Using Potent NorA Efflux Pump Inhibitors Immobilized on Nanometric Alpha-Zirconium Phosphate to Reduce Biofilm Formation
Source: Materials (Basel). 2021 Feb 1;14(3):670. doi: 10.3390/ma14030670 (PMC7867184; doi:10.3390/ma14030670)
Supplement: Supplementary file 1 [file materials-14-00670-s001.pdf]

materials

Supporting information

Biofunctionalization of Poly(lactide-*co*-glycolic acid) Using Potent NorA Efflux Pump Inhibitors Immobilized on Nanometric Alpha-Zirconium Phosphate to Reduce Biofilm Formation

Monica Pica [1], Nicla Messere [1], Tommaso Felicetti [1], Stefano Sabatini [1,*], Donatella Pietrella [2] and Morena Nocchetti [1,*]

[1] Dipartimento di Scienze Farmaceutiche, Università di Perugia, Via del Liceo, 1, 06123 Perugia, Italy; monica.pica@unipg.it (M.P.); niclamessere1@gmail.com (N.M.); tommaso.felicetti90@gmail.com (T.F.)
[2] Dipartimento di Medicina, Università di Perugia, Piazzale Gambuli, 1, 06132 Perugia, Italy; donatella.pietrella@unipg.it
* Correspondence: stefano.sabatini@unipg.it (S.S.); morena.nocchetti@unipg.it (M.N.)

Preparation of the Composite of ZrP and Thioridazine (ZrP/TZ)

A 0.06 M solution of thioridazine hydrochloride was prepared by dissolving 50 mg of TZ in 2 mL of water. The solution was added to 2.5 g of ZrP gel, so that the TZ/ZrP molar ratio was about 0.17. The mixture was left under stirring at room temperature for 30 minutes, then in oven at 80 °C for 2 days. The gelatinous product was centrifuged, washed two times with water and finally dried at room temperature under vacuum, over P_2O_5.

Preparation of the PLGA Composite Film Containing ZrP/TZ

A PLGA composite film, containing about 20 wt% ZrP/TZ was prepared by using the same procedure reported in paragraph 2.6 of the main manuscript. The composite film was labeled as film K.

Characterization of the ZrP/TZ Composite Powder

Figure S1a shows the TG profile of ZrP/TZ. The weight loss between 200 and 1200 °C is higher than that expected for ZrP (≈ 6 wt%), indicating that a certain amount of TZ was taken up by ZrP. By considerino the general composition $Zr(HPO_4)_2 \cdot n TZ \cdot m H_2O$, the values of n and m, calculated from the TG curve, were 0.12 and 1.9, respectively.

In order to evaluate if TZ molecules were intercalated by TZ, XRPD analysis was also carried out and the corresponding pattern was reported in Figure S1b. The broad peak at about 4.8° 2θ, reasonably assigned to the interlayer distance (d ≈ 18.4 Å), suggests that TZ was intercalated to some extent.

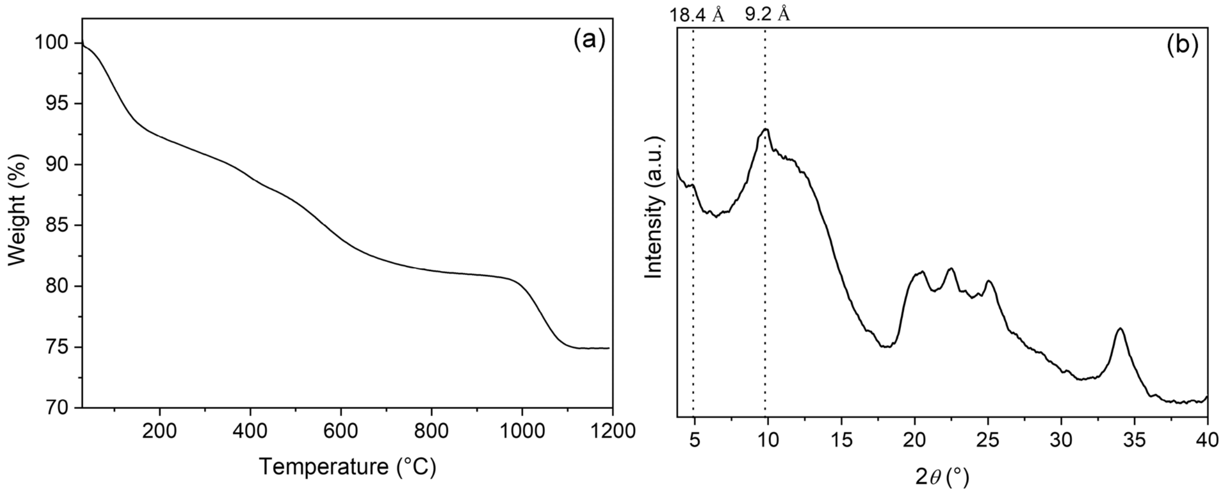

Figure S1. TG profile (**a**) and XRD (**b**) of ZrP/TZ.

Characterization of the PLGA_ZrP/TZ Composite Film

The TG profile and the XRD pattern of the PLGA film loaded with ZrP/TD (Film K) are shown in Figure S2.

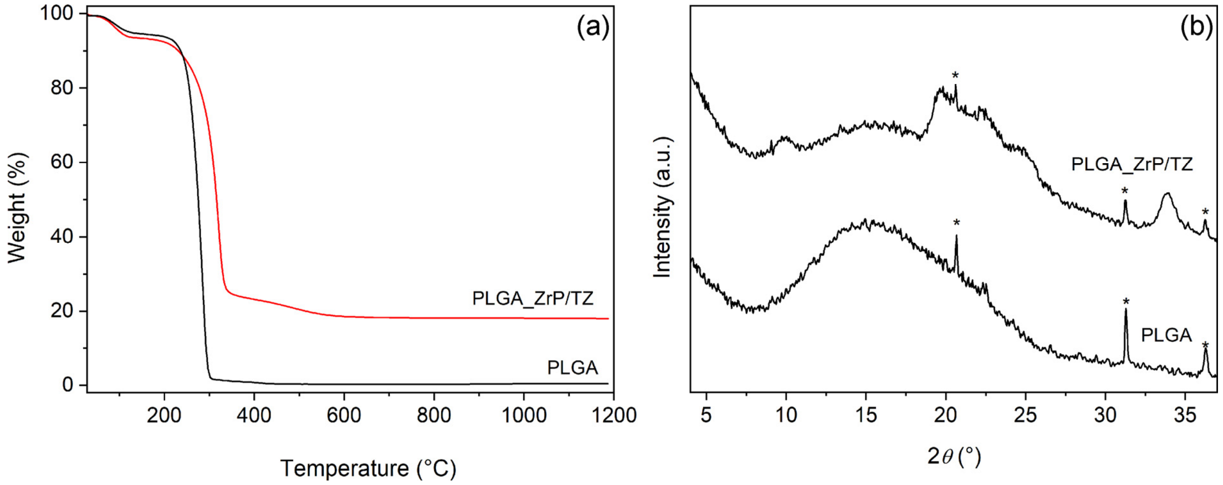

Figure S2. TG profile (**a**) and XRD (**b**) of PGLA (Film A) and PLGA_ZrPTZ (Film K).

The effective filler loading, calculated from the residual weight at 1200 °C of Figure S2a and due to ZrP_2O_7, was about 24.5%, corresponding to a 3 wt% of TZ in the composite film, while the XRD pattern of the composite film (Figure S2b) shows broad peaks at 10°, between 18° and 27°, and at 33.8° 2θ, ascribed to ZrP/TZ intercalation compound, suggesting the formation of a microcomposite.
